# Electroconvulsive seizures (ECS) do not prevent LPS-induced behavioral alterations and microglial activation

**DOI:** 10.1186/s12974-015-0454-x

**Published:** 2015-12-12

**Authors:** E. M. van Buel, F. J. Bosker, J. van Drunen, J. Strijker, W. Douwenga, H. C. Klein, U. L. M. Eisel

**Affiliations:** Department of Molecular Neurobiology, Groningen Institute of Evolutionary Life Sciences, University of Groningen, Nijenborgh 7, 9747 AG Groningen, Netherlands; Department of Psychiatry, University Medical Center Groningen, University of Groningen, Groningen, Netherlands; Department of Nuclear Medicine and Molecular Imaging, University Medical Center Groningen, University of Groningen, Groningen, Netherlands

**Keywords:** ECS, Depression-like behavior, LPS, Inflammation, Microglia

## Abstract

**Background:**

Long-term neuroimmune activation is a common finding in major depressive disorder (MDD). Literature suggests a dual effect of electroconvulsive therapy (ECT), a highly effective treatment strategy for MDD, on neuroimmune parameters: while ECT acutely increases inflammatory parameters, such as serum levels of pro-inflammatory cytokines, there is evidence to suggest that repeated ECT sessions eventually result in downregulation of the inflammatory response. We hypothesized that this might be due to ECT-induced attenuation of microglial activity upon inflammatory stimuli in the brain.

**Methods:**

Adult male C57Bl/6J mice received a series of ten electroconvulsive seizures (ECS) or sham shocks, followed by an intracerebroventricular (i.c.v.) lipopolysaccharide (LPS) or phosphate-buffered saline (PBS) injection. Brains were extracted and immunohistochemically stained for the microglial marker ionized calcium-binding adaptor molecule 1 (Iba1). In addition, a sucrose preference test and an open-field test were performed to quantify behavioral alterations.

**Results:**

LPS induced a short-term reduction in sucrose preference, which normalized within 3 days. In addition, LPS reduced the distance walked in the open field and induced alterations in grooming and rearing behavior. ECS did not affect any of these parameters. Phenotypical analysis of microglia demonstrated an LPS-induced increase in microglial activity ranging from 84 to 213 % in different hippocampal regions (CA3 213 %; CA1 84 %; dentate gyrus 131 %; and hilus 123 %). ECS-induced alterations in microglial activity were insignificant, ranging from −2.6 to 14.3 % in PBS-injected mice and from −20.2 to 6.6 % in LPS-injected mice.

**Conclusions:**

We were unable to demonstrate an effect of ECS on LPS-induced microglial activity or behavioral alterations.

**Electronic supplementary material:**

The online version of this article (doi:10.1186/s12974-015-0454-x) contains supplementary material, which is available to authorized users.

## Background

Electroconvulsive therapy (ECT) is a highly effective clinical treatment strategy for major depressive disorder (MDD). Multiple studies have shown superior efficacy for ECT compared to pharmaceutical treatment strategies [1]. Over the past decades, numerous studies have been published that have attempted to unravel the neurobiological mechanisms behind the antidepressant effects of ECT. These studies have resulted in a rapid increase in the understanding of these mechanisms, with one of the most consistent findings being a strong effect on neurogenesis accompanied by increased levels of neurotrophic factors [2–16]. Nevertheless, many questions remain unanswered.

Long-term neuroimmune activation is a common finding in MDD. MDD is associated with elevated levels of pro-inflammatory cytokines and decreased levels of anti-inflammatory cytokines [17]. In addition, there is evidence for altered functionality of immune cells, including increased activity of microglia, the resident immune cells of the brain [18]. Literature suggests that antidepressant treatment may, at least in part, normalize neuroimmune changes associated with MDD [19–21].

Although literature on the effects of ECT on neuroimmune parameters is relatively scarce, the available literature points towards a dual effect: while ECT acutely increases inflammatory parameters, repeated ECT sessions eventually result in a downregulation of the inflammatory response [22–25]. For example, although acute ECT upregulates serum levels of pro-inflammatory cytokines such as TNFα, IL-1β, and IL-6 in MDD patients [22–24], serum TNFα levels gradually decrease over the course of multiple ECT sessions until they eventually reach levels within the range of healthy control subjects [22]. In addition, animal studies have provided evidence for acute transient microglia activation upon electroconvulsive seizures (ECS; an animal model for ECT) [26].

Based on these findings, we hypothesized that repeated ECT sessions suppress the activation of resident immune cells upon inflammatory stimuli in the brain. This might explain the downregulation of inflammatory parameters and clinical improvement as observed in successfully treated MDD patients. To test this hypothesis, we exposed mice that had received a series of ECS to a pro-inflammatory stimulus (an intracerebroventricular lipopolysaccharide injection) inducing depressive-like behavior. We assessed the extent of microglia activation in the hippocampus upon this inflammatory stimulus with an ionized calcium-binding adaptor molecule 1 (Iba1) immunohistochemical staining. In addition, behavioral tests were performed to investigate whether ECS could prevent behavioral alterations associated with inflammation.

## Methods

### Animals

Three-month-old male C57Bl/6J mice were obtained from Janvier. Animals were housed in individual cages under standardized conditions (temperature 21 ± 2 °C, humidity 50–60 %, 12:12 h light/dark cycle). Food and water were available ad libitum. Animals were handled for at least 1 week prior to the start of the experiment. Throughout the experiment, body weight was measured on a daily basis. The Ethical Committee for the Use of Experimental Animals of the University of Groningen had approved all experiments (DEC6229G).

### Experimental design

**Fig. 1:**
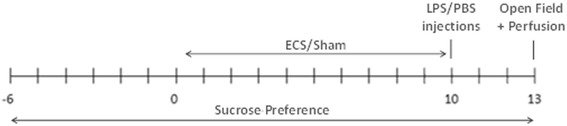
Experimental design. Daily ECS/sham procedures were performed for 10 days, followed by one single LPS or PBS injection. Three days after the LPS or PBS injection, an open-field test was performed and the mice were sacrificed. A sucrose preference test was started 6 days prior to the first ECS/sham procedure and continued for the entire duration of the experiment

Figure 1 shows the experimental design that was followed during this study. Mice (*n* = 40) were equally divided across four groups (PBS + sham, PBS + ECS, LPS + sham, and LPS + ECS). A sucrose preference test was started 6 days prior to the first ECS treatment and was continued throughout the experiment. ECS or sham ECS was performed daily for 10 days. Lipopolysaccharide (LPS) or phosphate-buffered saline (PBS) injections were delivered 1 day after the last ECS/sham treatment. Three days after the LPS/PBS injections, an open-field test was performed and mice were sacrificed. In previous experiments, this timepoint was associated with maximal neuroinflammation [27].

### Electroconvulsive seizures

Mice received a series of one electroconvulsive or sham treatment per day for 10 days. Mice were anesthetized with sevoflurane, and electroconvulsive shocks were delivered via ear clip electrodes using a pulse generator (Ugo Basile, Comerio, Italy), set at the following shock parameters: 80 mA, 50 Hz, 1 s duration, and 0.5 ms pulse width. All mice developed tonic-clonic seizures lasting approximately 10–15 s, which is comparable to the seizure duration induced by electroconvulsive seizures (ECS) in other experiments [28–30]. The total duration of anesthesia was approximately 5 min per mouse. Mice in the sham groups underwent the same procedure, including sevoflurane anesthesia and placement of the ear clip electrodes, without delivery of the electroconvulsive shock.

### Intracerebroventricular LPS injections

Mice were anesthetized with sevoflurane and fixated in a stereotact. Analgesia was provided via a subcutaneous injection of 2.5 mg/kg finadyne. A skin incision was made to expose the skull, and holes were drilled perpendicularly to the skull. Mice were injected with 1 μl PBS or 1 μl 5 mg/ml LPS (l-6529, serotype 055:B5, Sigma-Aldrich) in PBS at the following coordinates: −2.5 mm dorsal/ventral, −1.0 mm lateral, and −0.5 mm anterior/posterior from bregma. Injections were performed at a constant rate of 0.3 μl/min using a syringe pump (TSE Systems, Bad Homburg, Germany) in combination with a 25-μl Hamilton syringe connected to a 1-μl Hamilton needle (cat. nr.170431, Omnilabo). The needle was not removed from the brain until 5 min after the injection to prevent any leakage of LPS via the injection canal. The holes were sealed with dental cement, and the incision was closed with sutures.

### Sucrose preference test

Mice were given a choice between two drinking pipettes, one containing normal drinking water and the other containing a 0.5 % sucrose solution. Mice had access to both pipettes 24 h per day. Water and sucrose intake were measured on a daily basis. The relative location of both pipettes was switched on a daily basis to avoid place preference. Mice that showed such a place preference and therefore failed to develop a consistent preference for sucrose were excluded from subsequent analysis.

### Open-field test

Mice were placed in a round arena with a diameter of 50 cm consisting of two zones: a border zone adjacent to the wall of the arena and an inner zone in the center of the arena. Mice were allowed to explore their surroundings freely for 7 min. All experiments were recorded by an overhead camera. Distance moved and time spent in the different zones were scored using EthoVision software (Noldus Information Technology, Wageningen, Netherlands). An observer blinded to the treatment scored grooming and rearing behavior manually.

### Iba1 immunohistochemistry

Mice (*n* = 6 per group) were transcardially perfused with a saline solution containing 0.5 % heparin followed by a 4 % paraformaldehyde (PFA) solution in 0.1 M phosphate buffer. Brains were removed and postfixated for 24 h in 4 % PFA in 0.1 M PBS, followed by 18 h in a 30 % sucrose solution for cryoprotection. Brains were frozen using liquid nitrogen and cut into sections of 20 μm. A DAB staining for the microglial marker Iba1 was performed on free-floating hippocampal and prefrontal cortex (PFC) sections. Sections were incubated for 72 h with primary antibody (rabbit anti-Iba1, Wako Chemicals, Neuss, Germany) diluted 1:2500 in 0.01 M PBS (pH 7.4) containing 1 % bovine serum albumin (BSA) and 0.1 % triton X-100. Subsequently, sections were incubated with secondary antibody (1:500; goat anti-rabbit, Jackson ImmunoResearch, Suffolk, UK) for 2 h, followed by a 1-h incubation with avidin-biotin complex (1:500; Vector Laboratories Burlingame, CA, USA). Finally, 0.075 mg/ml DAB was added and the DAB reaction was initiated with 100 μl 0.1 % H_2_O_2_. Sections were carefully rinsed with 0.01 M PBS between all steps of the protocol, and all antibodies and chemicals were diluted in 0.01 M PBS unless stated otherwise.

Microglial activation was measured with Image-Pro software (Image-Pro Plus 6.0.0.26, Media Cybernetics, Inc. Rockville, USA) using a method described by Hovens et al. [31]. In short, photographs were taken at ×100 magnification. The software first automatically determined the total coverage of the microglia cells (cell bodies and processes). Subsequently, intensity and size thresholds were changed in such a way that only the coverage of the cell bodies was measured. The cell bodies to total coverage ratio was calculated. This ratio was shown to accurately reflect activation state in previous experiments [31]. In addition, the number of cells was counted manually.

### Statistical analysis

All statistical analyses were performed using GraphPad Prism 5.0 (GraphPad Software, San Diego, CA, USA). Most data was statistically analyzed using a two-way ANOVA to calculate *p* values for the main effects of group (LPS/PBS) and treatment (ECS/sham) and the group × treatment interaction effect. Body weight was analyzed via a repeated measures ANOVA with post hoc Bonferroni test. Data are presented as mean ± standard error.

## Results

### LPS decreases body weight

**Fig. 2:**
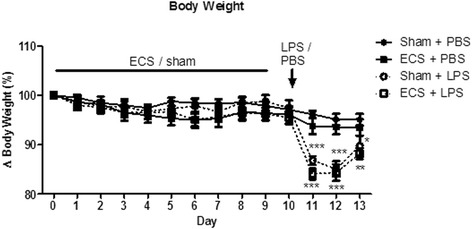
Body weight. Body weight is presented as a percentage of baseline body weight (measured prior to the first ECS session on day 0). ECS/sham sessions were performed daily from day 0 to day 9. I.c.v. LPS/PBS injections were performed on day 10. *p < 0.05; **p < 0.01; ***p < 0.001 compared to sham + PBS

Figure 2 shows body weight as a percentage of baseline body weight (measured on day 0 prior to the first ECS/sham session; see also Additional file 1). Body weight was not significantly influenced by ECS treatment. However, intracerebroventricular (i.c.v.) LPS injections resulted in a marked decrease in body weight in both the ECS and sham groups. Body weight partly normalized 3 days after LPS injection. This is in line with previous experiments that showed that body weight is decreased up to 3 days after LPS injection [27]. I.c.v. PBS injection did not result in significant alterations in body weight.

### LPS acutely decreases sucrose preference

**Fig. 3:**
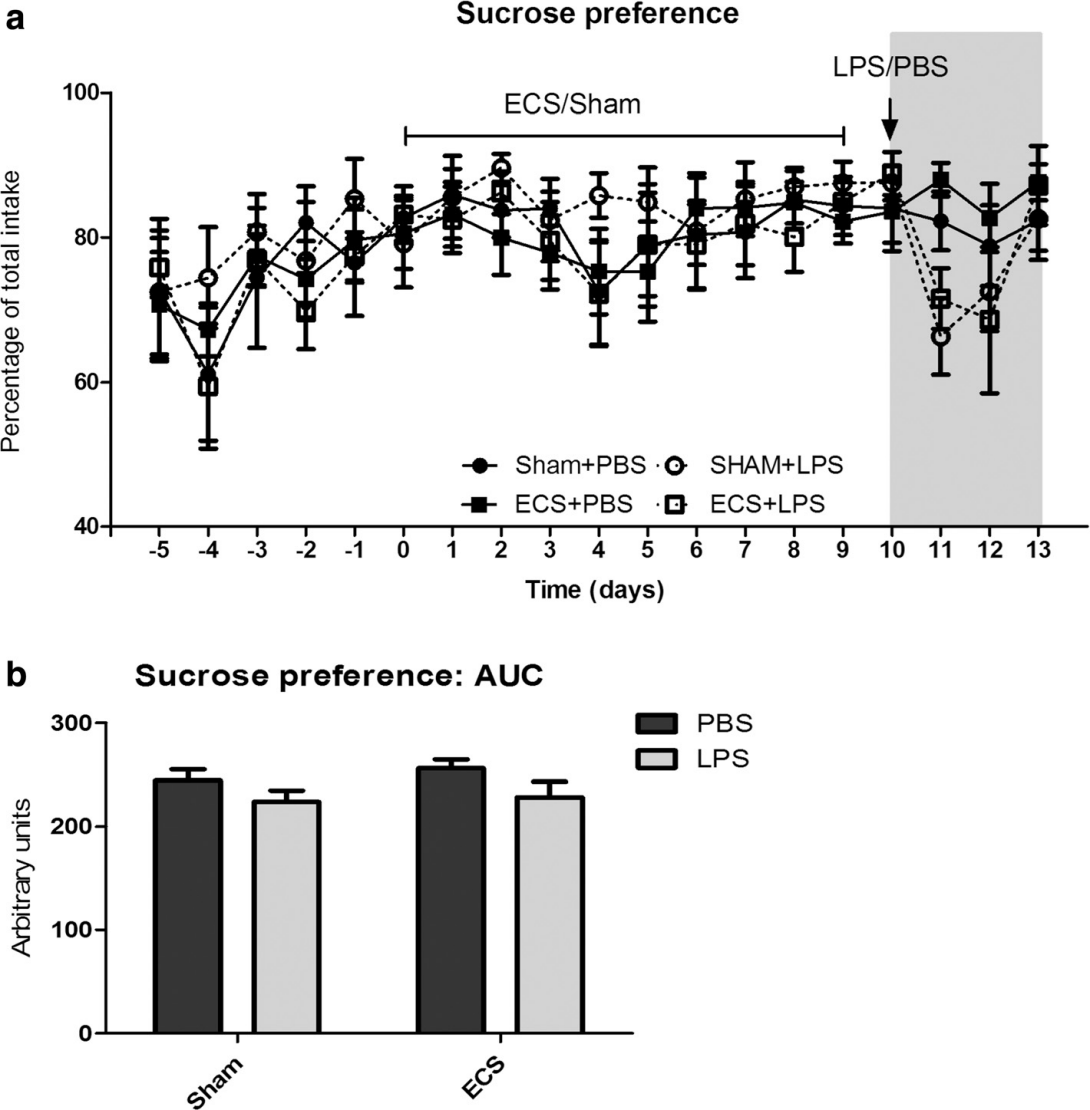
Sucrose preference. **a** Sucrose preference is presented as a percentage of total fluid intake over 24 h. ECS/sham sessions were performed daily from day 0 to day 9. I.c.v. LPS/PBS injections were performed on day 10. **b** An area under the curve (AUC) was calculated for days 10–13 (the *gray area* in **a**). There was a significant main effect for group (PBS/LPS; *p* = 0.0405) but not for treatment (sham/ECS) or group × treatment interaction (two-way ANOVA)

Figure 3a shows sucrose preference over the entire duration of the experiment (see also Additional file 2). During the habituation phase prior to the start of the ECS sessions, sucrose preference gradually increased to around 80 %. Sucrose preference was not influenced by electroconvulsive seizures. I.c.v. LPS injections caused a temporary decrease in sucrose preference in both the ECS and sham groups, which fully normalized 3 days after injection. An area under the curve (Fig. 3b, Additional file 2) was calculated for the time between LPS injection and the end of the experiment (days 10–13; the gray area in a). The area under the curve (AUC) was analyzed via a two-way ANOVA, demonstrating a significant main effect for group (LPS/PBS; *p* = 0.0405). No significant effects were found for treatment or treatment × group interaction (*p* = 0.7372 and *p* = 0.4808, respectively).

### Both LPS and ECS influence behavior in the open-field test

**Fig. 4:**
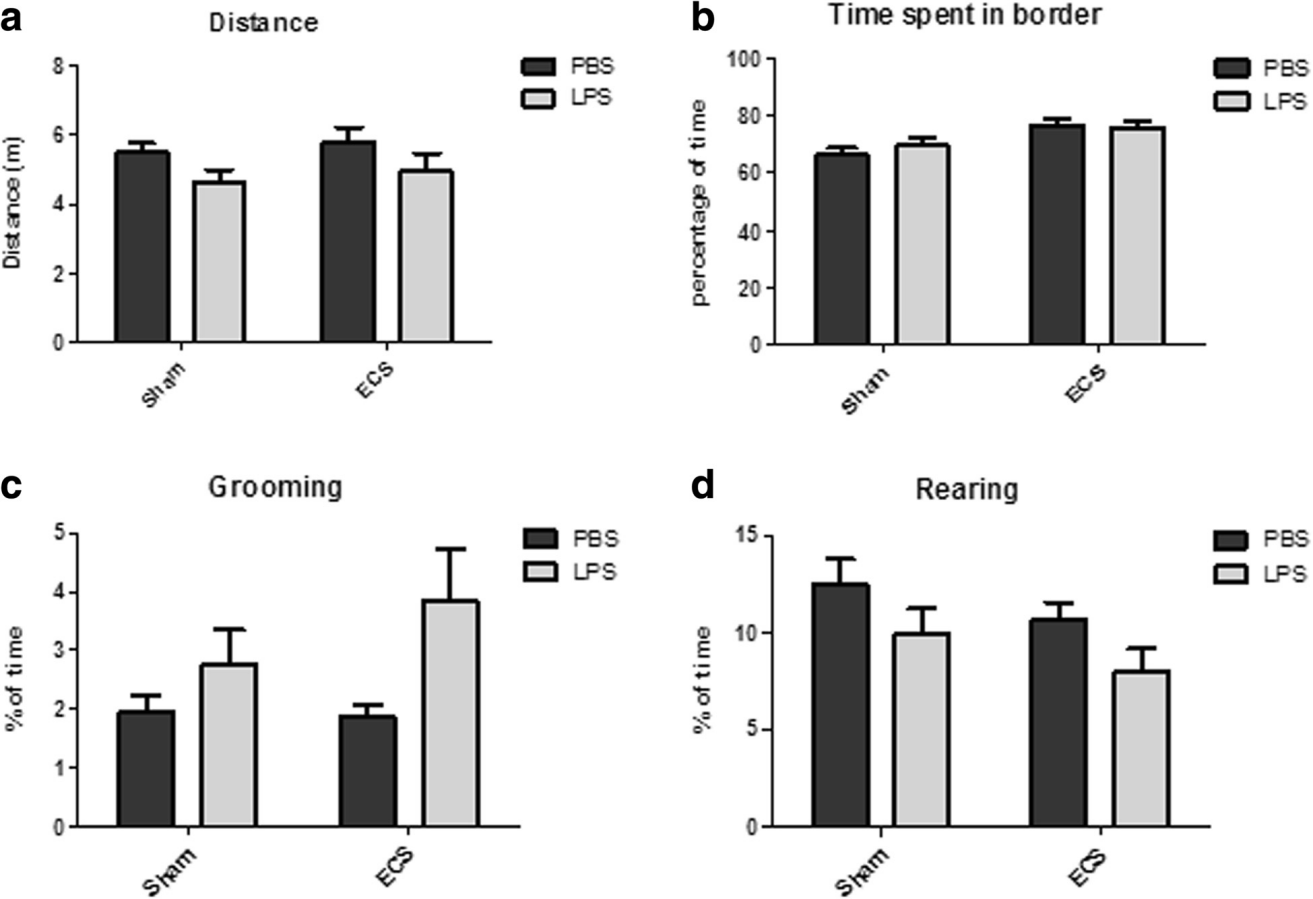
Open-field test. Three days after PBS/LPS injection, an open-field test was performed. The graphs represent **a** distance moved during the test, **b** percentage of time spent in the border zone of the arena, **c** percentage of time spent grooming, and **d** percentage of time spent rearing. There was a significant main effect for group (PBS/LPS) on the distance moved during the test (**a**; *p* = 0.0361), percentage of time spent grooming (**b**; *p* = 0.0334), and percentage of time spent rearing (**d**; *p* = 0.0159). In addition, a significant main effect for treatment (sham/ECS) was found for the percentage of time spent in the border zone (**b**; *p* = 0.005). Other main effects did not reach significance, and interaction effects were not significant for any of these parameters

Distance moved and time spent in the border zone of the arena (Fig. 4a, b; Additional file 3) were scored automatically. LPS decreased the total distance moved during the test regardless of electroconvulsive seizures (main effect for group: *p* = 0.0361). No significant effects were observed for sham/ECS treatment and treatment × group interaction (*p* = 0.4525 and *p* = 0.9956, respectively).

The time spent in the border zone was significantly increased in the ECS groups (main effect for sham/ECS treatment: *p* = 0.005). No significant differences were observed for the PBS/LPS group effect and the treatment × group interaction effect (*p* = 0.6323 and *p* = 0.4739, respectively).

Grooming and rearing behavior (Fig. 4c, d; Additional file 3) were scored manually. In both the ECS and sham groups, LPS increased grooming behavior (main effect for group: *p* = 0.0334) but decreased rearing behavior (main effect for group: *p* = 0.0159). The main effect for sham/ECS treatment and the treatment × group interaction effect were not significant for either behavior (grooming: *p* = 0.3702 and *p* = 0.2830, respectively; rearing: *p* = 0.1126 and *p* = 0.9521, respectively).

### ECS does not prevent LPS-induced microglial activity

The depression-like behavioral effects of LPS are believed to be related to their inflammatory effects. Here, we were interested in the effect of ECS on LPS-induced inflammatory changes in the brain. Therefore, we measured the ratio of the microglia cell bodies to the total coverage of cell bodies and processes. As activated microglia undergo a conformational change towards shorter and thicker processes and increased size of the cell bodies, this ratio reflects the level of microglial activity [31].

**Fig. 5:**
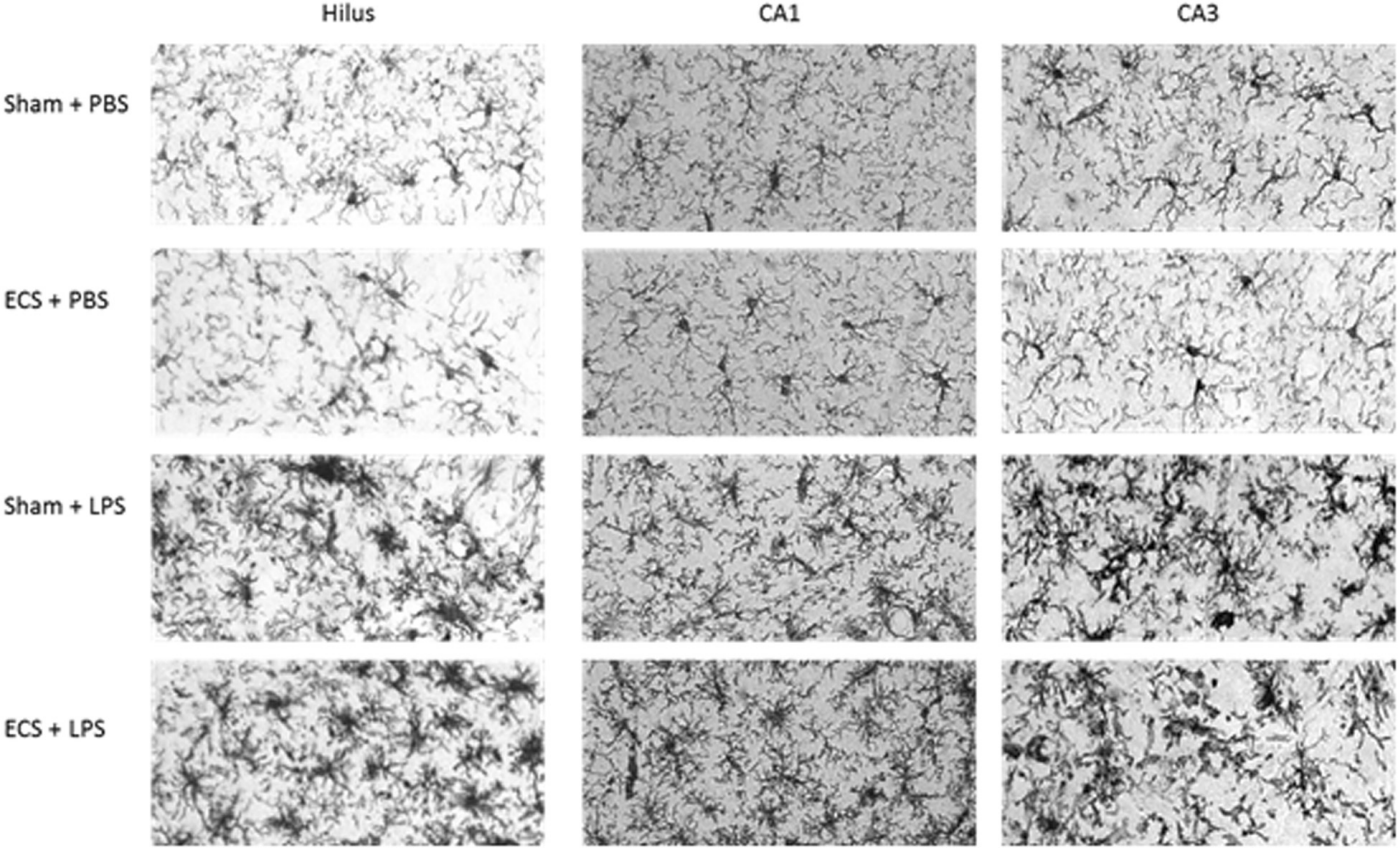
Iba1 immunohistochemistry. Three days after PBS/LPS injection, mice were sacrificed, and brain sections were immunohistochemically stained for the microglial marker Iba1. This figure shows representative pictures of Iba1 stained microglia for three hippocampal areas (hilus, CA1, and CA3) and four treatment groups (sham + PBS, ECS + PBS, sham + LPS, and ECS + LPS)

**Fig. 6:**
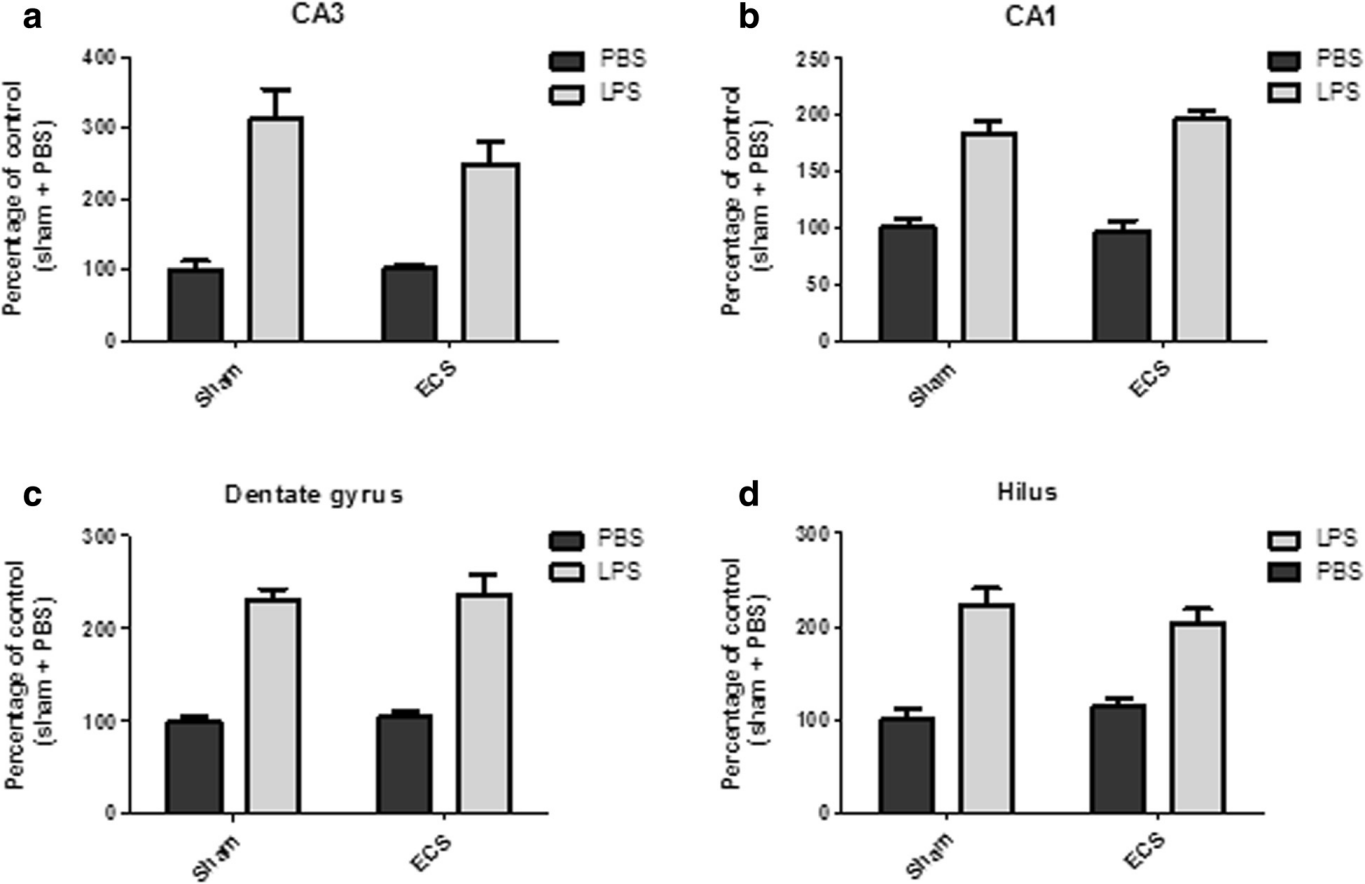
Microglia activation. Microglia activation is reflected by the ratio of the coverage of the cell bodies to the total coverage (cell bodies and processes). Data are given as percentages of the sham + PBS group. **a** Microglia activation in the CA3 area of the hippocampus. **b** Microglia activation in the CA1 area of the hippocampus. **c** Microglia activation in the hippocampal dentate gyrus. **d** Microglia activation in the hippocampal hilus. In all areas, a highly significant main effect for group (LPS/PBS) was found (*p* < 0.0001 for all areas). The main effects for treatment (sham/ECS) and group × treatment interaction effects did not reach significance for any of the analyzed brain areas

Representative pictures of Iba1-stained microglia in the hilus, CA1, and CA3 regions of the hippocampus are shown in Fig. 5. Figure 6 presents microglial activity in the different hippocampal areas (see also Additional file 4). In the sham groups, LPS increased microglial activity by 84 to 213 % in different hippocampal areas (CA3 213 %; CA1 84 %; dentate gyrus 131 %; and hilus 123 %). ECS altered microglial activity by −2.6 to 14.3 % in PBS-injected mice (CA3 1.6 %; CA1 −2.6 %; dentate gyrus 5.4 %; and hilus 14.3 %) and by −20.2 to 6.6 % in LPS-injected mice (CA3 −20.2 %; CA1 6.6 %; dentate gyrus 2.6 %; hilus −8.8 %; compared to the sham + LPS group). In all areas that were analyzed (CA1, CA3, hilus, and dentate gyrus), there was a highly significant main effect for group (*p* < 0.0001 for all areas) but not for treatment (CA3: *p* = 0.2708; CA1: *p* = 0.6038; dentate gyrus: *p* = 0.6610; hilus: *p* = 0.8269). The group × treatment interaction effect was not significant for any of the hippocampal regions (CA3: *p* = 0.2468; CA1: *p* = 0.4246; dentate gyrus: *p* = 0.9815; hilus: *p* = 0.2318).

**Fig. 7:**
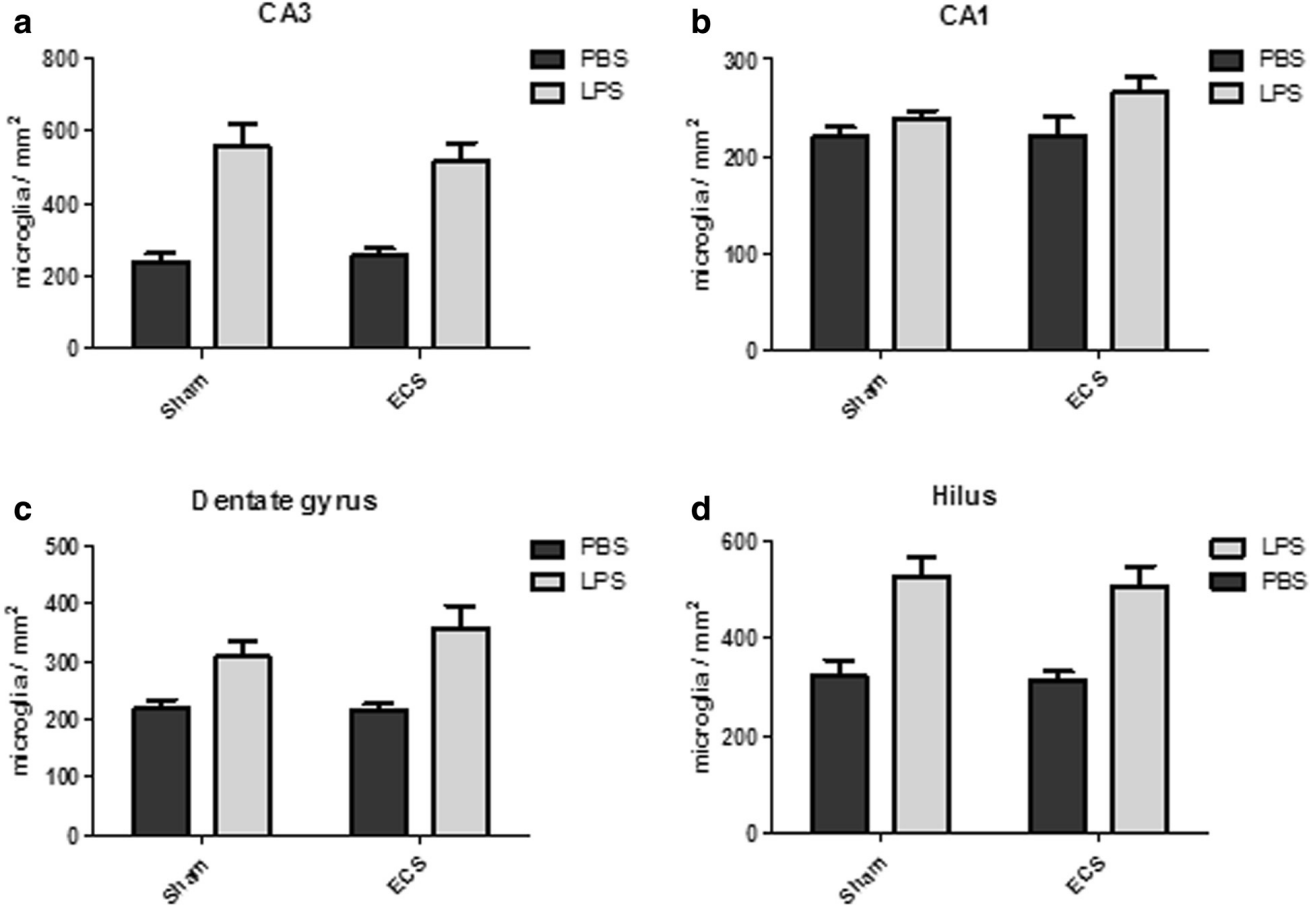
Quantification of microglia. Microglia were counted manually. The graphs show the number of microglia per square millimeter in the following hippocampal areas: CA3 (**a**), CA1 (**b**), dentate gyrus (**c**), and hilus (**d**). In all areas, a significant main effect for group (LPS/PBS) was found (*p* < 0.0001 for the CA3 and the hilus, *p* = 0.0336 for the CA1, and *p* = 0.0002 for the dentate gyrus). The main effects for treatment (sham/ECS) and group × treatment interaction effects did not reach significance for any of the analyzed brain areas

In addition to microglial activity, the number of microglia was counted in each hippocampal area (Fig. 7, Additional file 4). LPS induced an increase in the number of microglia in all hippocampal areas (main effect for LPS/PBS: *p* < 0.0001 for the CA3 and the hilus, *p* = 0.0336 for the CA1, and *p* = 0.0002 for the dentate gyrus). Again, ECS did not affect the number of microglia and there was no significant interaction effect (CA3: *p* = 0.7831 and *p* = 0.5466, respectively; CA1: *p* = 0.3027 and *p* = 0.3606, respectively; dentate gyrus: *p* = 0.3508 and *p* = 0.3046, respectively; hilus: *p* = 0.6494 and *p* = 0.8480, respectively).

## Discussion

The goal of this study was to investigate whether repeated ECS sessions suppress the activation of microglia after an inflammatory challenge of the brain. We showed that ECS does not significantly reduce behavioral effects nor microglial activation after an LPS challenge. In contrast, literature indicates that effective repeated ECT treatment in patients may result in normalization of serum levels of cytokines such as TNF-alpha [22, 32]. The dose of LPS was chosen based on its ability to induce depression-like behavior in previous experiments [27]. However, the extreme microglial activation observed in the LPS groups suggests that the inflammatory effects of LPS in this study may not be comparable to the inflammatory alterations in MDD patients, which are possibly relatively mild and could have a more prolonged nature [33]. Therefore, we cannot exclude that the inflammatory and behavioral effect of LPS injection might be too strong to be significantly reduced by ECS treatment.

One should also keep in mind that the mechanisms involved in the relatively short-term inflammatory response induced by LPS might differ substantially from those involved in the more long-term, low-grade inflammation associated with MDD. Therefore, the nature of the inflammatory response in this study may be quite different from the nature of inflammation associated with clinical depression. Indeed, although there is a substantial overlap in the physiological parameters associated with MDD and LPS-induced depression-like behavior, there are also several differences, for example in the relative contribution of certain types of cytokines [34]. Although previous experiments in the same department had demonstrated depression-like behavior up to 4 days after i.c.v. LPS injection [27], the sucrose preference data presented in this study suggest that depression-like behavior normalizes within 3 days after LPS injection. This inconsistency might be due to methodological differences. While Dobos et al. [27] assessed depression-like behavior via a forced swim test; we have chosen to perform a sucrose preference test as this test allows for repeated measurements.

Most LPS studies have focused on the short-term effects of LPS on behavior (several hours to 2 days after injection); several studies have investigated longer-term effects of LPS, although these studies have employed peripheral intraperitoneal LPS injections instead of central injections. The majority of these studies indicate that the effects of LPS on depressive-like behavior in the sucrose preference test [35], forced swim test [36, 37], or tail suspension test [38] are transient in healthy adult rodents, normalizing within 24–72 h. These studies are in line with the results of this study, demonstrating decreased sucrose preference that normalizes within 72 h. Painsipp et al. [39], however, have found increased immobility in the forced swim test and decreased sucrose preference up to 4 weeks after LPS injection in C57Bl/6 mice but not CD-1 mice. This shows that the effects of LPS may be dependent upon the animal and strain used in the experiment. In addition, differences in the serotype and concentration of LPS used for injection may have contributed to the discrepancies between these studies.

Several attempts have been made to induce a more chronic type of inflammation in rodents. For example, recent studies demonstrate that repeated peripheral LPS injections in rodents can induce microglial activation, prolonged depression-like behavior, and expression of a specific pattern of cytokines [40–42]. Although determination of the optimal dose and frequency of LPS injection requires further research, this method of inducing inflammation may be more comparable to the pathophysiology of depression. In addition, stress-related depression models, such as the chronic unpredictable mild stress model and the chronic social defeat stress model, are well known to increase the levels of certain inflammatory parameters, including pro-inflammatory cytokines and microglial activity [43–45]. It would thus be interesting to study the effects of ECS on inflammatory parameters in such models.

It is important to note that in this study, we have investigated the ability of ECS to suppress the reactivity of microglial cells towards inflammatory stimuli. Thus, we have given ECS prior to LPS injection. Therefore, we cannot draw conclusions regarding the ability of ECS to reduce microglial activity in case of pre-existing neuroimmune activation. Thus, it is possible ECS may reduce pre-existing neuroimmune activation without affecting microglial reactivity to inflammatory stimuli administered post-ECS.

The involvement of microglia in MDD pathophysiology is confirmed by a recent PET study investigating translocator protein (TSPO) binding as a biomarker for microglial activity in MDD patients with an active major depressive episode [46]. Microglial activity was found to be increased in various brain areas, including the hippocampus. Moreover, this increase correlated with the severity of depression.

## Conclusions

With the read out parameters chosen here, this study suggests that ECS does not have a suppressive effect on microglia activation in an inflammatory depression model, which is possibly due to the rather powerful challenge with intracerebral LPS injection. This does not rule out that inflammatory states in milder depression models might be effectively reduced by ECS.

## Additional files

Additional file 1: Table S1. Presents body weight data per day over the course of the experiment. (PDF 169 kb) Additional file 2: Table S2. Shows sucrose preference data per day, presented as a ratio of sucrose intake compared to total fluid intake. In addition, data are shown for the AUC for day 10-13 of the experiment. (PDF 174 kb) Additional file 3: Table S3. Summarizes the distance moved in the open field, as well as the percentage of time spent in each zone and the percentage of time spent rearing and grooming. (PDF 168 kb) Additional file 4: Table S4. Presents data of microglial activity measurements (ratio cell bodies : total coverage) and microglia cell counts in different hippocampal areas (hilus, dentate gyrus, CA3, and CA1). (PDF 181 kb)

## Abbreviations

AUC: area under the curve
BSA: bovine serum albumin
ECS: electroconvulsive seizures
ECT: electroconvulsive therapy
Iba1: ionized calcium-binding adaptor molecule 1
LPS: lipopolysaccharide
MDD: major depressive disorder
PBS: phosphate-buffered saline
PFA: paraformaldehyde
PFC: prefrontal cortex
TSPO: translocator protein

## References

[1] UK ECT Review Group (2003). Efficacy and safety of electroconvulsive therapy in depressive disorders: a systematic review and meta-analysis. Lancet.

[2] Malberg JE, Eisch AJ, Nestler EJ, Duman RS (2000). Chronic antidepressant treatment increases neurogenesis in adult rat hippocampus. J Neurosci.

[3] Duman RS, Vaidya VA (1998). Molecular and cellular actions of chronic electroconvulsive seizures. J ECT.

[4] Nibuya M, Morinobu S, Duman RS (1995). Regulation of BDNF and trkB mRNA in rat brain by chronic electroconvulsive seizure and antidepressant drug treatments. J Neurosci.

[5] Altar CA, Whitehead RE, Chen R, Wortwein G, Madsen TM (2003). Effects of electroconvulsive seizures and antidepressant drugs on brain-derived neurotrophic factor protein in rat brain. Biol Psychiatry.

[6] Angelucci F, Aloe L, Jimenez-Vasquez P, Mathe AA (2002). Electroconvulsive stimuli alter the regional concentrations of nerve growth factor, brain-derived neurotrophic factor, and glial cell line-derived neurotrophic factor in adult rat brain. J ECT.

[7] Kyeremanteng C, James J, Mackay J, Merali Z (2012). A study of brain and serum brain-derived neurotrophic factor protein in Wistar and Wistar-Kyoto rat strains after electroconvulsive stimulus. Pharmacopsychiatry.

[8] Haghighi M, Salehi I, Erfani P, Jahangard L, Bajoghli H, Holsboer-Trachsler E (2013). Additional ECT increases BDNF-levels in patients suffering from major depressive disorders compared to patients treated with citalopram only. J Psychiatr Res.

[9] Piccinni A, Del Debbio A, Medda P, Bianchi C, Roncaglia I, Veltri A (2009). Plasma brain-derived neurotrophic factor in treatment-resistant depressed patients receiving electroconvulsive therapy. Eur Neuropsychopharmacol.

[10] Marano CM, Phatak P, Vemulapalli UR, Sasan A, Nalbandyan MR, Ramanujam S (2007). Increased plasma concentration of brain-derived neurotrophic factor with electroconvulsive therapy: a pilot study in patients with major depression. J Clin Psychiatry.

[11] Bocchio-Chiavetto L, Zanardini R, Bortolomasi M, Abate M, Segala M, Giacopuzzi M (2006). Electroconvulsive therapy (ECT) increases serum brain derived neurotrophic factor (BDNF) in drug resistant depressed patients. Eur Neuropsychopharmacol.

[12] Segi-Nishida E, Warner-Schmidt J, Duman RS (2008). Electroconvulsive seizure and VEGF increase the proliferation of neural-stem-like cells in rat hippocampus. Proc Natl Acad Sci.

[13] Minelli A, Zanardini R, Abate M, Bortolomasi M, Gennarelli M, Bocchio-Chiavetto L (2011). Vascular endothelial growth factor (VEGF) serum concentration during electroconvulsive therapy (ECT) in treatment resistant depressed patients. Prog Neuropsychopharmacol Biol Psychiatry.

[14] Gwinn RP, Kondratyev A, Gale K (2002). Time-dependent increase in basic fibroblast growth factor protein in limbic regions following electroshock seizures. Neuroscience.

[15] Kondratyev A, Ved R, Gale K (2002). The effects of repeated minimal electroconvulsive shock exposure on levels of mRNA encoding fibroblast growth factor-2 and nerve growth factor in limbic regions. Neuroscience.

[16] Conti G, Gale K, Kondratyev A (2009). Immunohistochemical evaluation of the protein expression of nerve growth factor and its TrkA receptor in rat limbic regions following electroshock seizures. Neurosci Res.

[17] Dantzer R, O’Connor JC, Freund GG, Johnson RW, Kelley KW (2008). From inflammation to sickness and depression: when the immune system subjugates the brain. Nat Rev Neurosci.

[18] Beumer W, Gibney SM, Drexhage RC, Pont-Lezica L, Doorduin J, Klein HC (2012). The immune theory of psychiatric diseases: a key role for activated microglia and circulating monocytes. J Leukoc Biol.

[19] Hiles SA, Baker AL, de Malmanche T, Attia J (2012). Interleukin-6, C-reactive protein and interleukin-10 after antidepressant treatment in people with depression: a meta-analysis. Psychol Med.

[20] Hannestad J, DellaGioia N, Bloch M (2011). The effect of antidepressant medication treatment on serum levels of inflammatory cytokines: a meta-analysis. Neuropsychopharmacology.

[21] Walker FR (2013). A critical review of the mechanism of action for the selective serotonin reuptake inhibitors: do these drugs possess anti-inflammatory properties and how relevant is this in the treatment of depression?. Neuropharmacology.

[22] Hestad KA, Tonseth S, Stoen CD, Ueland T, Aukrust P (2003). Raised plasma levels of tumor necrosis factor alpha in patients with depression: normalization during electroconvulsive therapy. J ECT.

[23] Lehtimaki K, Keranen T, Huuhka M, Palmio J, Hurme M, Leinonen E (2008). Increase in plasma proinflammatory cytokines after electroconvulsive therapy in patients with depressive disorder. J ECT.

[24] Fluitman SB, Heijnen CJ, Denys DA, Nolen WA, Balk FJ, Westenberg HG (2011). Electroconvulsive therapy has acute immunological and neuroendocrine effects in patients with major depressive disorder. J Affect Disord.

[25] van Buel EM, Patas K, Peters M, Bosker FJ, Eisel UL, Klein HC (2015). Immune and neurotrophin stimulation by electroconvulsive therapy: is some inflammation needed after all?. Transl Psychiatry.

[26] Jansson L, Wennstrom M, Johanson A, Tingstrom A (2009). Glial cell activation in response to electroconvulsive seizures. Prog Neuropsychopharmacol Biol Psychiatry.

[27] Dobos N, de Vries EF, Kema IP, Patas K, Prins M, Nijholt IM (2012). The role of indoleamine 2,3-dioxygenase in a mouse model of neuroinflammation-induced depression. J Alzheimers Dis.

[28] Sakaida M, Sukeno M, Imoto Y, Tsuchiya S, Sugimoto Y, Okuno Y (2013). Electroconvulsive seizure-induced changes in gene expression in the mouse hypothalamic paraventricular nucleus. J Psychopharmacol.

[29] Jinno S, Kosaka T (2008). Reduction of Iba1-expressing microglial process density in the hippocampus following electroconvulsive shock. Exp Neurol.

[30] Steward O, Kelley MS, Schauwecker PE (1997). Signals that regulate astroglial gene expression: induction of GFAP mRNA following seizures or injury is blocked by protein synthesis inhibitors. Exp Neurol.

[31] Hovens IB, Nyakas C, Schoemaker RG (2014). A novel method for evaluating microglial activation using ionized calcium-binding adaptor protein-1 staining: cell body to cell size ratio. Neuroimmunology Neuroinflammation.

[32] Hestad KA, Tonseth S, Stoen CD, Ueland T, Aukrust P (2005). Inflammation and depression: further studies are needed. J ECT.

[33] Raison CL, Miller AH (2011). Is depression an inflammatory disorder?. Curr Psychiatry Rep.

[34] Dunn AJ, Swiergiel AH, de Beaurepaire R (2005). Cytokines as mediators of depression: what can we learn from animal studies?. Neurosci Biobehav Rev.

[35] Wang D, Lin W, Pan Y, Kuang X, Qi X, Sun H (2011). Chronic blockade of glucocorticoid receptors by RU486 enhances lipopolysaccharide-induced depressive-like behaviour and cytokine production in rats. Brain Behav Immun.

[36] Godbout JP, Moreau M, Lestage J, Chen J, Sparkman NL, O’Connor J (2008). Aging exacerbates depressive-like behavior in mice in response to activation of the peripheral innate immune system. Neuropsychopharmacology.

[37] Aguilar-Valles A, Kim J, Jung S, Woodside B, Luheshi GN (2014). Role of brain transmigrating neutrophils in depression-like behavior during systemic infection. Mol Psychiatry.

[38] Corona AW, Huang Y, O’Connor JC, Dantzer R, Kelley KW, Popovich PG (2010). Fractalkine receptor (CX3CR1) deficiency sensitizes mice to the behavioral changes induced by lipopolysaccharide. J Neuroinflammation.

[39] Painsipp E, Kofer MJ, Sinner F, Holzer P (2011). Prolonged depression-like behavior caused by immune challenge: influence of mouse strain and social environment. PLoS One.

[40] Kubera M, Curzytek K, Duda W, Leskiewicz M, Basta-Kaim A, Budziszewska B (2013). A new animal model of (chronic) depression induced by repeated and intermittent lipopolysaccharide administration for 4 months. Brain Behav Immun.

[41] Fischer CW, Elfving B, Lund S, Wegener G (2015). Behavioral and systemic consequences of long-term inflammatory challenge. J Neuroimmunol.

[42] Borges BC, Rorato R, Antunes-Rodrigues J, Elias LL (2012). Glial cell activity is maintained during prolonged inflammatory challenge in rats. Braz J Med Biol Res.

[43] Farooq RK, Isingrini E, Tanti A, Le Guisquet AM, Arlicot N, Minier F (2012). Is unpredictable chronic mild stress (UCMS) a reliable model to study depression-induced neuroinflammation?. Behav Brain Res.

[44] Pan Y, Chen XY, Zhang QY, Kong LD (2014). Microglial NLRP3 inflammasome activation mediates IL-1beta-related inflammation in prefrontal cortex of depressive rats. Brain Behav Immun.

[45] Reader BF, Jarrett BL, McKim DB, Wohleb ES, Godbout JP, Sheridan JF (2015). Peripheral and central effects of repeated social defeat stress: monocyte trafficking, microglial activation, and anxiety. Neuroscience.

[46] Setiawan E, Wilson AA, Mizrahi R, Rusjan PM, Miler L, Rajkowska G (2015). Role of translocator protein density, a marker of neuroinflammation, in the brain during major depressive episodes. JAMA Psychiatry.

