# Supplementary material for: Electroconvulsive seizures (ECS) do not prevent LPS-induced behavioral alterations and microglial activation
Source: J Neuroinflammation. 2015 Dec 12;12:232. doi: 10.1186/s12974-015-0454-x (PMC4676811; doi:10.1186/s12974-015-0454-x)
Supplement: Additional file 1: Table S1. — Presents body weight data per day over the course of the experiment. (PDF 169 kb) [file 12974_2015_454_MOESM1_ESM.pdf]

SI1: Body weight (percentage compared to day 0)

	Sham + PBS		ECS + PBS		Sham + LPS		ECS + LPS	
Day	Mean	Sem	Mean	Sem	Mean	Sem	Mean	Sem
1	99,52	0,61	98,58	1,25	98,77	0,80	98,00	1,05
2	98,46	0,49	98,22	1,55	97,73	1,23	97,54	1,16
3	97,98	0,76	96,46	1,60	97,72	1,47	96,53	0,97
4	97,43	0,72	95,97	1,56	96,74	1,37	96,44	1,20
5	98,73	0,81	95,28	1,38	97,29	1,51	96,66	1,13
6	98,48	0,76	95,05	1,70	97,82	1,62	95,04	1,36
7	98,34	0,84	95,29	1,71	96,71	1,23	95,70	1,06
8	98,57	0,96	96,61	1,68	98,51	1,20	96,38	1,25
9	97,88	0,91	96,40	1,51	98,73	1,31	96,27	1,28
10	97,08	0,70	96,07	1,71	97,50	1,46	95,45	1,31
11	96,17	0,69	93,79	1,63	86,80	0,89	84,06	1,10
12	95,12	1,10	93,57	1,49	85,09	1,54	84,34	1,70
13	95,08	1,19	93,48	1,71	89,77	2,07	88,18	1,11
