# Supplementary material for: Electroconvulsive seizures (ECS) do not prevent LPS-induced behavioral alterations and microglial activation
Source: J Neuroinflammation. 2015 Dec 12;12:232. doi: 10.1186/s12974-015-0454-x (PMC4676811; doi:10.1186/s12974-015-0454-x)
Supplement: Additional file 2: Table S2. — Shows sucrose preference data per day, presented as a ratio of sucrose intake compared to total fluid intake. In addition, data are shown for the AUC for day 10-13 of the experiment. (PDF 174 kb) [file 12974_2015_454_MOESM2_ESM.pdf]

SI2: Sucrose preference (ratio intake sucrose solution : total fluid intake)

	Sham + PBS		ECS + PBS		Sham + LPS		ECS + LPS	
**Day**	Mean	Sem	Mean	Sem	Mean	Sem	Mean	Sem
-5	72,76	9,84	70,64	7,35	72,43	8,57	75,83	4,09
-4	61,12	9,20	67,23	3,62	74,45	7,01	59,44	8,63
-3	74,44	9,67	77,42	4,24	80,78	5,25	76,94	3,51
-2	82,06	5,06	74,22	5,31	76,76	8,19	69,85	5,25
-1	76,51	7,36	79,67	5,88	85,40	5,52	77,43	3,36
0	82,73	3,10	80,59	4,95	79,14	6,05	83,19	3,88
1	85,94	3,60	83,22	4,46	85,59	5,74	82,46	4,59
2	83,79	3,76	80,00	5,17	89,65	1,92	86,23	2,92
3	84,03	2,34	77,93	5,12	82,44	5,70	79,53	5,38
4	72,44	7,21	75,30	5,92	85,83	3,09	72,19	7,15
5	78,94	8,48	75,27	6,94	84,93	4,79	79,03	7,17
6	80,39	4,19	84,04	4,30	80,88	8,06	79,03	6,02
7	80,76	6,37	84,18	3,29	85,33	5,11	81,94	5,78
8	85,25	3,99	84,85	2,88	87,01	2,68	80,10	4,84
9	84,39	4,06	82,21	3,02	87,54	3,00	84,97	2,76
10	84,07	5,93	83,64	4,34	87,57	2,40	88,88	2,99
11	82,29	4,05	88,02	2,32	66,34	5,26	71,55	4,20
12	78,87	5,61	82,73	4,73	72,50	5,51	68,58	10,10
13	82,43	4,25	87,68	2,50	82,60	5,73	87,24	5,47

AUC day 10-13

Group	Mean	Sem
Sham + PBS	244,41	10,61
ECS + PBS	256,41	8,31
Sham + LPS	223,92	10,72
ECS + LPS	233,60	14,56
