# Supplementary material for: Electroconvulsive seizures (ECS) do not prevent LPS-induced behavioral alterations and microglial activation
Source: J Neuroinflammation. 2015 Dec 12;12:232. doi: 10.1186/s12974-015-0454-x (PMC4676811; doi:10.1186/s12974-015-0454-x)
Supplement: Additional file 3: Table S3. — Summarizes the distance moved in the open field, as well as the percentage of time spent in each zone and the percentage of time spent rearing and grooming. (PDF 168 kb) [file 12974_2015_454_MOESM3_ESM.pdf]

SI3: Open field test

Distance (m)		
Group	Mean	Sem
Sham + PBS	5,50	0,27
ECS + PBS	5,80	0,41
Sham + LPS	4,66	0,34
ECS + LPS	4,95	0,52

Time spent in border (%)		
Group	Mean	Sem
Sham + PBS	66,23	2,52
ECS + PBS	76,37	2,78
Sham + LPS	69,52	3,00
ECS + LPS	75,71	2,49

Grooming (%)		
Group	Mean	Sem
Sham + PBS	1,95	0,28
ECS + PBS	1,85	0,24
Sham + LPS	2,74	0,61
ECS + LPS	3,83	0,88

Rearing (%)		
Group	Mean	Sem
Sham + PBS	12,49	1,30
ECS + PBS	10,65	0,89
Sham + LPS	9,96	1,26
ECS + LPS	7,98	1,19
