# Supplementary material for: Electroconvulsive seizures (ECS) do not prevent LPS-induced behavioral alterations and microglial activation
Source: J Neuroinflammation. 2015 Dec 12;12:232. doi: 10.1186/s12974-015-0454-x (PMC4676811; doi:10.1186/s12974-015-0454-x)
Supplement: Additional file 4: Table S4. — Presents data of microglial activity measurements (ratio cell bodies : total coverage) and microglia cell counts in different hippocampal areas (hilus, dentate gyrus, CA3, and CA1). (PDF 181 kb) [file 12974_2015_454_MOESM4_ESM.pdf]

SI4: Microglial activity and number of cells

Microglial activity (ratio cell bodies : total coverage; percentage compared to sham+PBS group)

CA3		
Group	Mean	Sem
Sham + PBS	100,0	12,8
ECS + PBS	101,6	6,3
Sham + LPS	313,4	41,7
ECS + LPS	250,2	31,8

CA1		
Group	Mean	Sem
Sham + PBS	100,0	8,4
ECS + PBS	97,4	9,7
Sham + LPS	184,3	10,6
ECS + LPS	196,4	7,2

DG		
Group	Mean	Sem
Sham + PBS	100,0	5,9
ECS + PBS	105,4	5,8
Sham + LPS	230,7	11,5
ECS + LPS	236,8	21,5

Hilus		
Group	Mean	Sem
Sham + PBS	100,0	10,9
ECS + PBS	114,3	9,1
Sham + LPS	223,0	17,2
ECS + LPS	203,4	16,1

Number of microglia / square mm

CA3		
Group	Mean	Sem
Sham + PBS	240,80	22,47
ECS + PBS	255,52	22,92
Sham + LPS	557,23	65,43
ECS + LPS	517,93	49,49

CA1		
Group	Mean	Sem
Sham + PBS	219,85	10,57
ECS + PBS	221,55	19,40
Sham + LPS	238,61	7,91
ECS + LPS	266,40	15,07

DG		
Group	Mean	Sem
Sham + PBS	218,18	14,80
ECS + PBS	215,72	9,88
Sham + LPS	307,29	26,44
ECS + LPS	357,54	38,56

Hilus		
Group	Mean	Sem
Sham + PBS	322,45	33,32
ECS + PBS	313,34	18,70
Sham + LPS	529,62	37,42
ECS + LPS	507,28	42,23
